# Role of PECAM-1 in radiation-induced liver inflammation

**DOI:** 10.1111/jcmm.12630

**Published:** 2015-07-14

**Authors:** Ihtzaz Ahmed Malik, Ina Stange, Gesa Martius, Silke Cameron, Margret Rave-Fränk, Clemens Friedrich Hess, Volker Ellenrieder, Hendrik Andreas Wolff

**Affiliations:** a Department of Gastroenterology and Gastrointestinal Oncology, University Medical Center GoettingenGoettingen, Germany; b Department of Radiotherapy and Radiooncology, University Medical Center GoettingenGoettingen, Germany

**Keywords:** liver, cytokines, adhesion molecules, radiation

## Abstract

Platelet endothelial cell adhesion molecule-1 (PECAM-1, CD31) is known to play an important role in hepatic inflammation. Therefore, we investigated the role of PECAM-1 in wild-type (WT) and knock-out (KO)-mice after single-dose liver irradiation (25 Gy). Both, at mRNA and protein level, a time-dependent decrease in hepatic PECAM-1, corresponding to an increase in intercellular cell adhesion molecule-1 (ICAM-1) (6 hrs) was detected in WT-mice after irradiation. Immunohistologically, an increased number of neutrophil granulocytes (NG) (but not of mononuclear phagocytes) was observed in the liver of WT and PECAM-1-KO mice at 6 hrs after irradiation. The number of recruited NG was higher and prolonged until 24 hrs in KO compared to WT-mice. Correspondingly, a significant induction of hepatic tumour necrosis factor (TNF)-α and CXC-chemokines (KC/CXCL1 interleukin-8/CXCL8) was detected together with an elevation of serum liver transaminases (6–24 hrs) in WT and KO-mice. Likewise, phosphorylation of signal transducer and activator of transcription-3 (STAT-3) was observed in both animal groups after irradiation. The level of all investigated proteins as well as of the liver transaminases was significantly higher in KO than WT-mice. In the cell-line U937, irradiation led to a reduction in PECAM-1 in parallel to an increased ICAM-1 expression. TNF-α-blockage by anti-TNF-α prevented this change in both proteins in cell culture. Radiation-induced stress conditions induce a transient accumulation of granulocytes within the liver by down-regulation/absence of PECAM-1. It suggests that reduction/lack in PECAM-1 may lead to greater and prolonged inflammation which can be prevented by anti-TNFα.

## Introduction

The recruitment of leucocytes from the blood circulation into the tissue is an important process in acute and chronic inflammation/tissue damage. This process is tightly regulated 1,2. Neutrophil granulocytes (NG) are the first line of immune defence among the leucocyte population entering into the stressed or inflamed tissue. In classical inflammation processes, inflammatory mediators like cytokines and chemokines are released from stressed or inflamed tissue. Their release results in the activation and the recruitment of leucocytes to the respective tissue, from the blood circulation 3,4. Indeed, a massive recruitment of leucocytes is observed in several clinical settings 4–6. This is directly linked to more tissue damage and an increased release of inflammatory mediators, ultimately leading to uncontrolled inflammation and reduced wound healing 4,7. To prevent uncontrolled inflammation, the balance of pro- and anti-inflammatory responses is crucial.

The process of leucocyte recruitment is divided into several steps, namely tethering, rolling, adhesion and crawling. The heterophilic interaction for leucocyte transendothelial migration takes place between molecules on the leucocyte and on the endothelial cell 8,9.

The major adhesion molecules involved in this process are selectins 10, integrins 8, vascular cell adhesion molecule-1, intercellular cell adhesion molecule-1 (ICAM-1), 11, ICAM-2 12, junctional adhesion molecules 13, VE-cadherin 50 and platelet endothelial cell adhesion adhesion molecule-1 (PECAM-1, CD31) 14. Among them, a significant role of the PECAM-1 in neutrophil transmigration has been proposed 8.

PECAM-1 (CD31) is a 130-kD glycoprotein that is expressed at the junctions of all continuous endothelia, as well as most bone marrow-derived hematopoietic cells, including platelets, monocytes, granulocytes and some lymphocytes 9,15. However, the role of PECAM-1 within the different steps of inflammation has been disputed.

Some earlier reports suggested the redistribution of PECAM-1 away from the intercellular junction of endothelial cells in response to pro-inflammatory cytokines. It suggests that spatial distribution of PECAM-1 has a regulatory role in the inflammatory environment 16. Accordingly, it has been described that PECAM-1 is not up-regulated transcriptionally 17.

In fact, a number of previous studies showed that PECAM-1 is down-regulated after granulocyte 18 and T cell activation 19. Additionally, the inhibition of PECAM-1 by antibodies in human monocytes showed induction of pro-inflammatory cytokines 20. Accordingly, in PECAM-1 deficient mice, an exaggerated inflammatory response was observed in different models of inflammation 21–23 and after endotoxic shock 24,25. This was attributed to a higher release of pro-inflammatory cytokines, an increased number of leucocytes and myeloperoxidase (MPO) activity 25.

Previous studies of our group showed an increase in tumour necrosis factor (TNF)-α, interleukin (IL)-6, interferon (IFN)-γ and ICAM-1 with a parallel decrease in PECAM-1 preceding the recruitment of inflammatory cells into the liver tissue in a classic inflammation rat model (CCI_4_) 26,27. Furthermore, we illustrated that the cytokines TNF-α as well as IFN-γ were able to down-regulate PECAM-1 (CD31) gene expression in parallel to ICAM-1 up-regulation in liver sinusoidal cells (endothelial cells and tissue macrophages) 26,28 and peripheral blood leucocytes 29.

Similarly, irradiation of human umbilical vein endothelial cells suggested the involvement of PECAM-1 in radiation-induced leucocyte transmigration 30. Moreover, in a whole lung radiation mouse model, endothelial cells of PECAM-1-deficient mice showed more apoptosis than the wild-type (WT) 31. Radiation-induced cell damage or death leads to leakage of cellular content into the adjacent tissue, resulting in the transmigration of inflammatory cells to the injured site.

We previously showed that irradiation of rat liver changed the gene expression of adhesion molecules and pro-inflammatory cyto- and chemokines, followed by recruitment of NG 32,33. However, the role of PECAM-1 in radiation-induced liver inflammation has not been studied so far.

Since the role of PECAM-1 as inflammation suppressor was described 25,34, we predict that PECAM-1 knock-out (KO)-mice are more sensitive to liver radiation. Our current data revealed that PECAM-1 KO mice showed an increased number of recruited NG which was accompanied by an increased release of inflammatory mediators (*e.g*. mainly TNF-α) and followed by elevation of liver transaminases compared to WT-mice after irradiation. Furthermore, anti-TNF-α treatment neutralized the radiation-induced down-regulation of PECAM-1 in cell culture. Our data support the role of PECAM-1 as negative regulator of radiation-induced inflammation in the liver.

## Material and methods

### Materials

All chemicals used were of analytical grade and purchased from commercial sources as follows: real-time PCR primers, M-MLV reverse transcriptase, reverse transcription buffer, 0.1 M dithiothreitol (DTT), Platinum SYBR green qPCR UDG mix were from Invitrogen (Carlsbad, CA, USA); dNTPs, Protector RNase inhibitor, Klenow enzyme, primer oligo (dT)15 for complementary DNA (cDNA) synthesis and Salmon sperm DNA were from Roche (Penzberg, Germany); Hybond N nylon membranes were purchased from Amersham Pharmacia Biotech (Amersham, UK), 4,6-diamidino-2-phenylindole (DAPI) from Southern Biotech (Birmingham, AL, USA). All other reagents and chemicals were from Sigma-Aldrich (St. Louis, MO, USA) or Merck (Darmstadt, Germany).

### Animal model

PECAM-1-KO (B6.129S-*Pecam1*^*Gt((OST16303)Lex*^/J) and WT (C57BL/6J) male mouse strains were purchased from Jackson Laboratory (Bar Harbor, Maine, USA). Two groups of male WT and PECAM-1 KO mice of 8-to-12-weeks old, about 20–28 g bw were irradiated selectively with the liver on focus with a single dose of 25 Gy (dose rate 2.4 Gy/min.) as described before 35. Sham-irradiated control animals were handled simultaneously. Treated animals and sham-irradiated controls were killed 1, 3, 6, 12, 24 and 48 hrs after irradiation. All animals received human care in accordance with the German Law for the Protection of Animals and the institutional guidelines. The local committee and responsible authority on animal welfare approved the treatment of the mice, and the experiments.

### Measurement of circulating alanine aminotransferase, aspartate aminotransferase and GLDH

At time points ranging from 1 to 48 hrs after targeted hepatic-irradiation, blood samples from the inferior vena cava were collected from sham-irradiated and irradiated mice and used for alanine aminotransferase (ALT) and aspartate aminotransferase (AST) measurement performed with analysis kits (DiaSys, Germany) as instructed. Glutamatdehydrogenase (GLDH) was also measured as instructed.

### RNA isolation and real-time PCR analysis

Total RNA from the livers of irradiated and sham-irradiated mice was isolated after homogenization in Trizol (Invitrogen) as described previously 36. For real-time PCR, reverse transcription of the extracted RNA samples was performed with a Superscript kit from Invitrogen. Briefly, cDNA was generated by reverse transcription of 1 μg of total RNA using 100 nM of dNTPs, 50 pM of primer oligo dT15, 200 U of moloney murine leukaemia virus reverse transcriptase (M-MLV RT), 16 U of protector RNase inhibitor in RT-buffer and 2.5 μl of 0.1 M DTT; real-time PCR was performed with a StepOnePlus^–^ sequence detection system (Applied Biosystems, Darmstadt, Germany) as described previously 36 with the primers shown in Table 1. Fold change expression was calculated using threshold cycle (Ct) values. The primers were synthesized by Invitrogen.

**Table 1:** Mice primer sequences used in this study

Primer	5–3 Forward	5–3 Reverse
PECAM-1	AACAGAAACCCGTGGAGATG	GTCTCTGTGGCTCTCGTTCC
ICAM-1	ATTCGTTTCCGGAGAGTGTG	CAGCACCGTGAATGTGATCT
TNF-α	CAAACCACCAAGTGGAGGAG	GTGGGTGAGGAGCACGTAGT
CXCL1	GGATTCACCTCAAGAACATCCAGAG	CACCCTTCTACTAGCACAGTGGTTG
CXCL8	GCTGGGATTCACCTCAAGAA	CTTTTGGACAATTTTCTGAACCA
β-Actin	ATTGTTACCAACTGGGACGACATG	CGAAGTCTAGAGCAACATAGCACA
GAPDH	AGAACATCATCCCTGCATCC	CACATTGGGGGTAGGAACAC

### Culture and treatment of the promonocytic cell line U-937

U-937 promonocytic cells were cultured and treated as described before 37. Briefly, U-937 promonocytic cells were seeded at 2 × 10^5^ cells/ml in RPMI 1640 medium containing 10% (v/v) heat-inactivated fetal calf serum, 2 mM/l L-glutamine, 1 mM/l sodium pyruvate and 1 mM of non-essential amino acids, and cultured at 37°C in an atmosphere of 95% air, 5% CO_2_. In a set of experiments, U-937 promonocytic cells were irradiated at 8 Gy and treated in the presence/absence of human TNF-α (10 ng/ml; Roche), or an antibody against human TNF-α infliximab (IFX; Remicade, 1000 μg/ml; MSD, Munich, Germany).

### Protein extraction and Western blot analysis

Protein extraction and Western blot analysis were performed as described before 29. Briefly, frozen tissue and cells were homogenized with an Ultra-turrax TP 18/10 (Cole-Parmer, Vernon Hills, IL, USA), three times for 10 sec. each, in 10 vol 50 mM TRIS-HCl buffer, pH 7.4, containing 150 mM sodium chloride, 1 mM EDTA, 1% Triton X-100, 1 mM phenylmethane sulfonyl-fluoride, 1 mM benzamidine, 1 mg/ml leupeptin, 10 mM chymostatin, 1 mg/ml antipain, and 1 mg/ml pepstatin A. The entire procedure was carried out at 4°C. Crude homogenates were passed five times through a 22G needle attached to a syringe and centrifuged for 5 min. at 10,000 × g at 4°C. The protein concentration was determined in supernatants using the bicinchoninic acid protein assay reagent kit (Pierce, Rockford, IL, USA). Aliquots of the homogenates were stored at −20°C until further used for Western blot analysis.

Samples of 50 μg proteins were applied per well and subjected to polyacrylamide gel electrophoresis using NuPAGE 4-12% Bis-Tris Gel (Invitrogen) under reducing conditions. After electrophoresis, the proteins were transferred to Hybond-enhanced chemiluminescence (ECL) nitrocellulose membranes. Immunodetection was performed according to the ECL Western blotting protocol described before 29. The antibodies and their concentration used for Western blotting in this study are mentioned in Table 2.

**Table 2:** Antibodies used in the study

Antibodies	Company	Reference number	WB	IHC
Rb to CK19	Abcam	Ab133496	–	1:100
Mouse anti-SMA	Sigma-Aldrich	A-2547	–	1:50
Rat anti F4/80 (BM8)	Abcam	Ab16911	–	1:10
Mouse anti Phospho-STAT3	Cell Signalling, Danvers, MA, USA	#4113S	1:1000	–
Beta-actin Ms anti ß-Actin Clone AC 15	Sigma-Aldrich	A-5441	1:5000	–
Rb to CD31(PECAM-1)	Abbiotec, San Diego, CA, USA	#250590	1:200	1:200
Rb anti-human MPO	DAKO, Hamburg, Germany	A0398	–	1:100
Rabbit pAb to ICAM-1	Abcam	Ab7815	1:1000	–

### Immunofluorescent double staining

Immunofluorescence staining was performed as described before 36. Briefly, for double-staining monoclonal F4/80 antibody was co-incubated with polyclonal antibody directed against cytokeratin 19 (CK19), polyclonal antibody directed against MPO with monoclonal antibody directed against alpha smooth muscle actin (α-SMA). Cryosections of 5 μm thickness and cells were fixed with cold acetone/methanol, washed in PBS and subsequently incubated with blocking medium (90% of a 0.1% bovine serum albumin, 10% fetal calf serum in PBS) for 1 hr at room temperature. The antibody dilutions were applied over night at 4°C onto the sections using a MPO polyclonal antibody (Abcam, Cambridge, UK) and F4/80 monoclonal antibody (Abcam), CK19 polyclonal antibody (Abcam) and α-SMA monoclonal antibody (Sigma-Aldrich), respectively. Non-immune serum was used as negative control. DAPI (4′,6-diamidino-2-phenylindole; Southern Biotech) served as nucleic acid stain. The slides were observed using an Axiovert 200M epifluorescence microscope (Zeiss, Jena, Germany). The antibodies used for immunofluorescence staining and their concentration in this study are mentioned in Table 2.

The number of positive cells (MPO, F4/80) was counted at each time-point from total liver and in the portal areas from 10 different portal vessels in the liver.

### Statistical analysis

The data were analysed using Graph pad Prism version 4 software (San Diego, CA, USA). All experimental errors are shown as SEM. Statistical significance was calculated by Student’s *t*-test. Significance was accepted at *P < 0.05.

## Results

### Effect of irradiation on the gene expression of PECAM-1 (CD31) and ICAM-1

**Figure 1:**
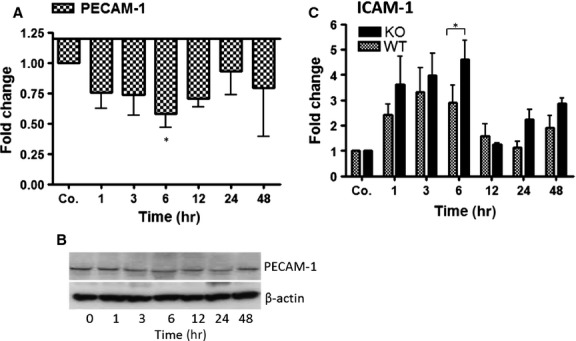
qRT-PCR and Western blot analysis from total RNA and total protein of the liver in WT and PECAM-1 KO mice after irradiation, respectively. Fold change in mRNA expression of (A) PECAM-1 and (C) ICAM-1 in irradiated mice liver at different time-points (1–48 hrs) related to sham-irradiated controls for each time-point in WT and PECAM-1 KO mice. qRT-PCR was normalized using two housekeeping genes: β-actin and GAPDH. Results represent mean value ± SEM of five animals *P < 0.05. (B) Western blot analysis of protein from mouse liver after irradiation using antibodies specific for PECAM-1 (upper). β-actin (lower) was used as a loading control. Results are representative of three to six experiments.

Total RNA extracted from the livers of irradiated and sham irradiated WT-mice was analysed by real-time PCR in WT and PECAM-1 KO mice compared to sham irradiated control mice livers. PECAM-1 (CD31) specific transcripts start decreasing early (1 hr) with a minimum at 6 hrs after liver irradiation (Fig. 1A) The results were confirmed at protein level by Western blot using specific antibody against PECAM-1 (CD31; Fig. 1B). In contrast, a parallel up-regulation of ICAM-1 transcript level with a maximum at 3–6 hrs was detected in the liver of WT after irradiation. Accordingly, an elevated gene expression of ICAM-1 was detected in PECAM-1 KO mice after irradiation. However, the magnitude of ICAM-1 was significantly higher at 6 hrs in PECAM-1 KO mice compared to WT mice after irradiation. The gene expression of ICAM-1 decreased afterwards (Fig. 1C).

The expression of PECAM-1 was not detected in the liver of sham irradiated and irradiated liver at any time-point in PECAM-1 KO mice (data not shown).

### Identification of recruited inflammatory cells by immunofluorescence double staining in the liver of irradiated mice

**Figure 2:**
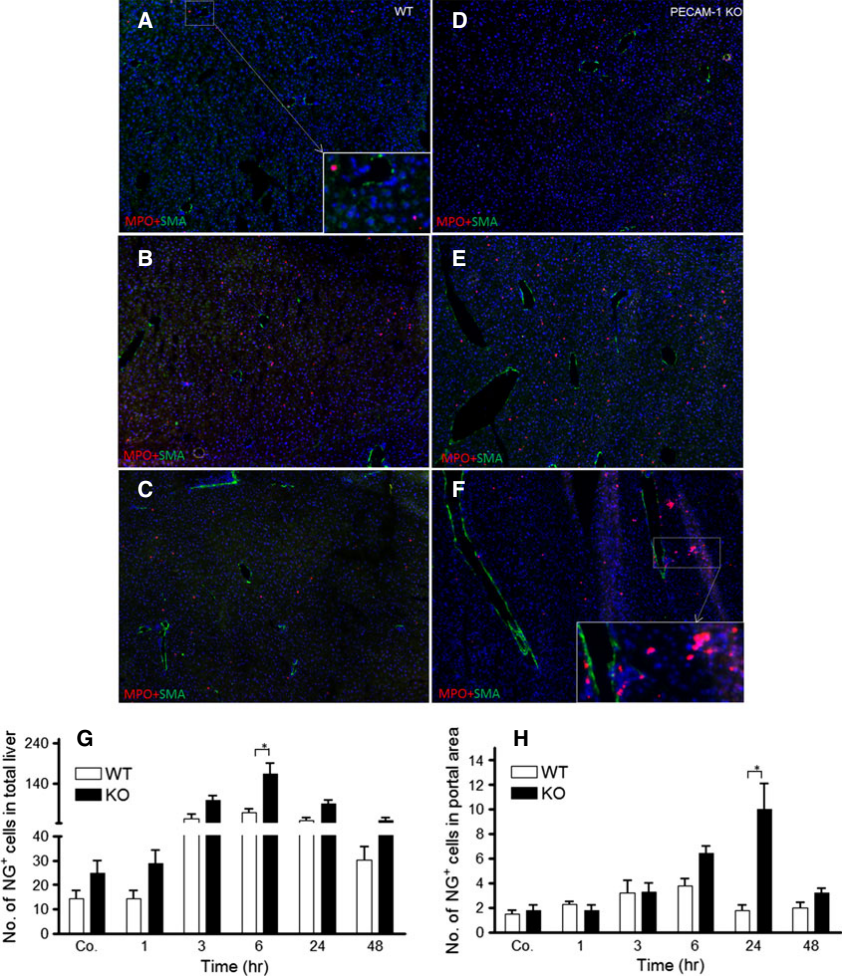
Double staining of liver sections with antibodies directed against α-SMA (green, a marker for myofibroblasts). MPO (red, a marker for granulocytes) followed by fluorescence immunodetection in sections of the liver of WT and PECAM-1 KO mice after irradiation (A and D) control liver (B and E) 6 hrs (C and F) and 24 hrs after irradiation. NG^+^ cells were also counted in total liver (G) and portal field (H) at different time points after mice liver irradiation (*P < 0.05, analysed by *t*-test). Results show representative pictures of six animals and six slides per time point. Original magnification, ×50.

Double immunofluorescence staining using antibodies against α-SMA (marker for myofibroblasts) and MPO (marker for granulocytes), detected an increased number of granulocytes (MPO^+^ cells) at 3 hrs with a maximum at 6 hrs around vessel walls but also throughout the liver of WT-mice after single-dose liver irradiation (Fig. 2A–C and G–H). The number of recruited granulocytes decreased at later time points (24 hrs) until the normal level. In comparison to sham irradiated WT mice, an increased number of MPO^+^ cells (14.3 ± 3.27) was detected in sham irradiated PECAM-1 KO (24.6 ± 5) mice. This increased number of granulocytes was further enhanced in PECAM-1 KO mice after irradiation. The number of granulocytes started to increase as early as in WT-mice, but the numbers of recruited cells in PECAM-1 KO was higher than in WT-mice at 6 hrs. Furthermore, the infiltration of MPO^+^ cells did not return to normal levels within 24 hrs but rather increased throughout the liver as well as around portal vessels in PECAM-1KO mice after irradiation (Fig. 2D–H). A mild increase around the vessel walls was visible in the liver tissue by α-SMA staining at 24 hrs in irradiated – compared to sham irradiated mice, and this increase was more intense in KO-mice than in WT mice.

**Figure 3:**
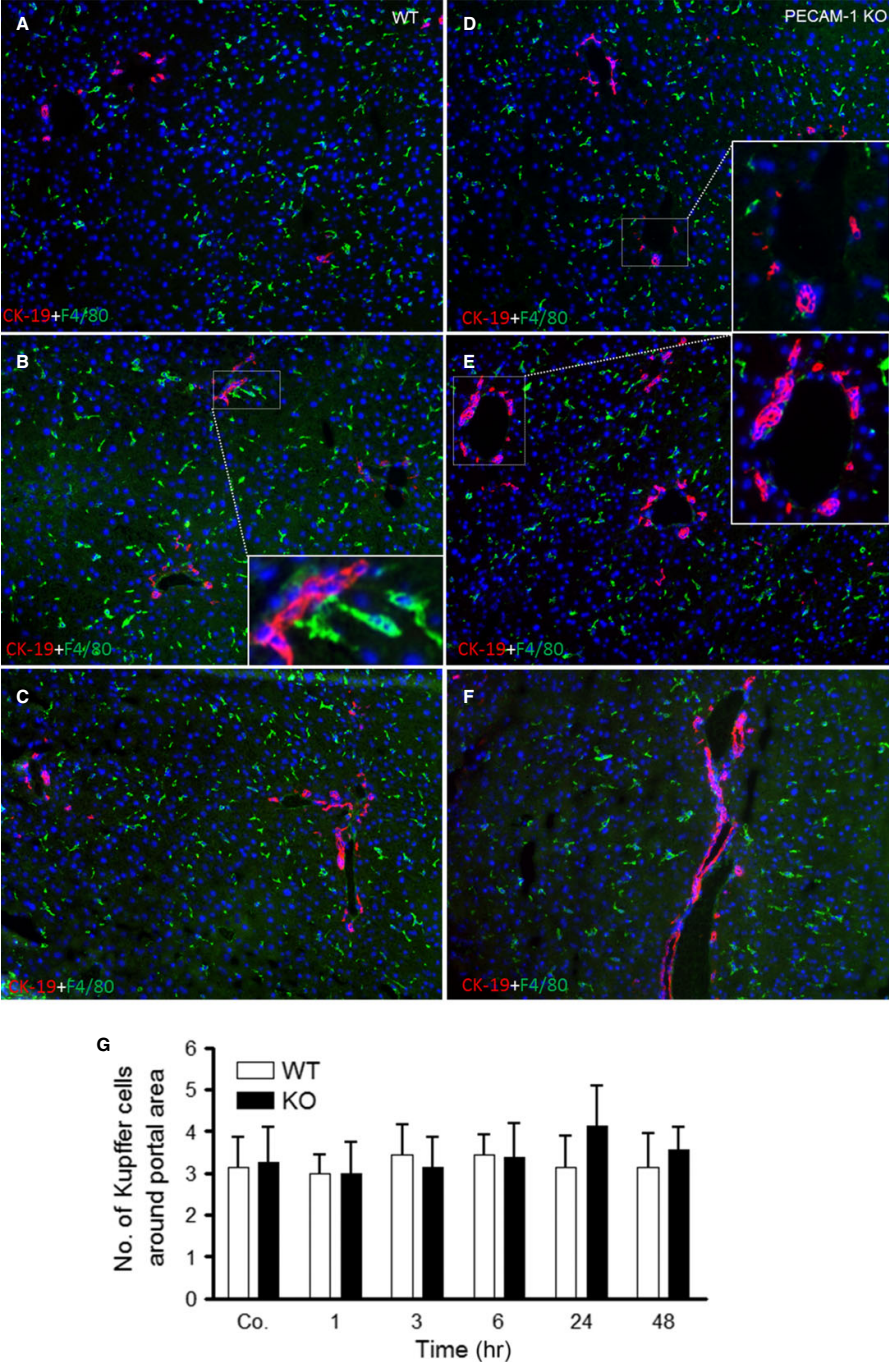
Double staining of liver sections with antibodies directed against CK-19 (red, a marker for bile duct cells), F4/80 (green, a marker for macrophages) followed by fluorescence immunodetection in sections of the liver of WT and PECAM-1 KO mice after irradiation. (A and D) control liver (B and E) 6 hrs (C and F) and 24 hrs after irradiation. (G) F4/80^+^ cells (macrophages) were also counted and no significant difference was observed at different time-points in mice liver after irradiation. Results show representative pictures of six animals and six slides per time-point. Original magnification, ×100.

Double staining using the antibodies against CK-19 (marker for bile duct cells) and F4/80 (marker for macrophages), F4/80^+^ cells showed an accumulation around the vessels but no increase in number of F4/80^+^ cells could be detected at any time-point after irradiation in the liver (Fig. 3A–G). However, the size of F4/80^+^ cells was increased after irradiation in WT and PECAM-1 KO mice (Fig. 3A–F). Furthermore, the size of CK-19^+^ cells increased in portal areas at 6–24 hrs in livers of both animal groups after irradiation (Fig. 3A–F).

### Kinetics of changes of proinflammatory mediators in acute mouse liver damage caused by irradiation

**Figure 4:**
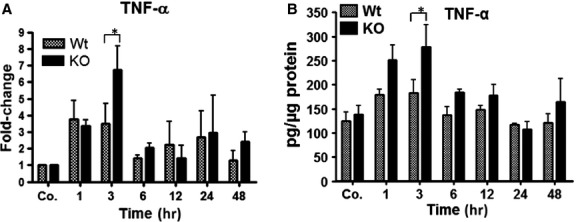
Total RNA and protein lysate analysis of TNF-α by qRT-PCR and ELISA, respectively, in the liver of WT and PECAM-1 KO mice after irradiation. The data are compared between WT and KO mice and with sham irradiated controls. (A) Fold change in mRNA expression of TNF-α, at different time points (1–48 hrs). RT-PCR was normalized to β-actin and GAPDH. (B) Liver tissue lysate TNF-α levels after irradiation. Values on the *y*-axis show the concentration of TNF-α measured with ELISA. Results represent mean ± SEM values of four to six experiments (in duplicate) compared with controls for each time point (*P < 0.05, analysed by *t*-test).

By means of RT-PCR, the gene expression of TNF-α showed a significant early (1 hr) increase, reaching a maximum (3.6 ± 1.2 and 6.7 ± 1.5-fold, respectively) at 3 hrs in WT and PECAM-1 KO mice compared to sham irradiated control mice livers (Fig. 4A). A similar trend was also measured by performing TNF-α ELISA (183 ± 27 pg/μg protein WT and 278 ± 45 pg/μg protein KO-mice) in liver tissue lysate (Fig. 4B). However, the magnitude of TNF-α increase in KO-mice was significantly higher than in WT-mice after irradiation both at RNA and protein level. A gradual decrease in TNF-α was observed thereafter in both animal groups (Fig. 4A and B).

**Figure 5:**
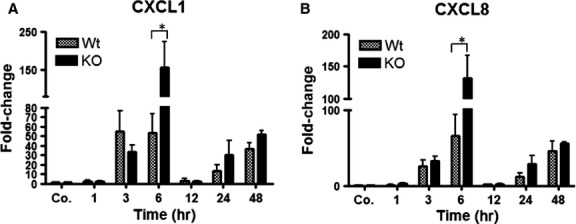
Fold change of mRNA expression of CXC-chemokines (CXCL1/KC and CXCL8/IL-8) in liver of wild-type and PECAM-1KO mice after liver irradiation. The data are compared between WT and KO mice and with sham irradiated controls. RT-PCR was normalized to β-actin. Results represent mean ± SEM values of four to six experiments (in duplicate) compared with controls for each time-point (*P < 0.05, analysed by *t*-test).

Correspondingly, a significant increase in both CXCL1/KC and CXCL8/IL-8 was detected at 3 hrs compared to sham irradiated controls both in KO and WT mice. This increase reached to its peak with a maximum at 6 hrs (CXCL1: 53 ± 19, 154 ± 67-folds, respectively, and CXCL8: 65 ± 28, 131 ± 35.8-folds, respectively) after irradiation in both animals groups in the liver. The magnitude of both chemokines was higher in KO than WT. The gene expression of both chemokines decreased afterwards in both KO and WT mice (Fig. 5A and B).

### Detection and change in CXCL1/KC in mice serum after liver irradiation

**Figure 6:**
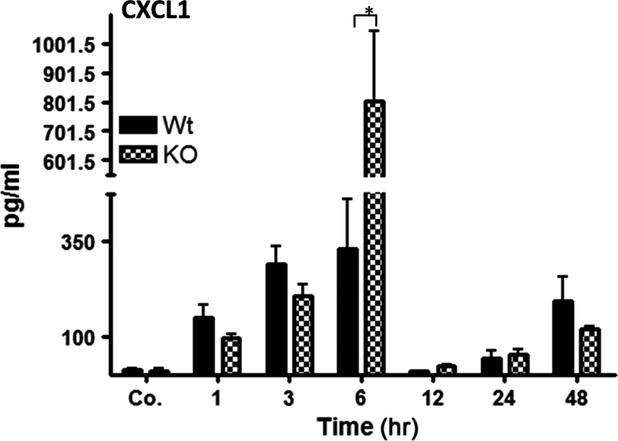
Serum CXCL1 levels in wild-type and PECAM-1KO mice after single-dose liver irradiation at different time points related to controls. The data are compared between WT and KO mice and with sham irradiated controls. Values on the *y*-axis show the serum concentration of CXCL1/KC measured with ELISA. These results are representative of six animals (statistically significant at *P < 0.05).

We measured the serum levels of CXCL1/KC after liver irradiation. A significant early increase in serum CXCL1/KC level at 3 hrs was detected in both WT and KO mice compared to sham irradiated controls. The maximum concentration of CXCL1/KC was found at 6 hrs followed by a quick decrease thereafter in both WT and KO-mice. The serum level of KO-mice was significantly higher (up to 802 ± 240 pg/ml) compared to WT-mice (up to 328 ± 131 pg/ml) at this time-point. A small rise in serum levels of CXCL1/KC was also observed at later time points which was higher in KO than WT after irradiation (Fig. 6).

### Kinetics of changes of liver enzyme serum levels after irradiation in mice

**Figure 7:**
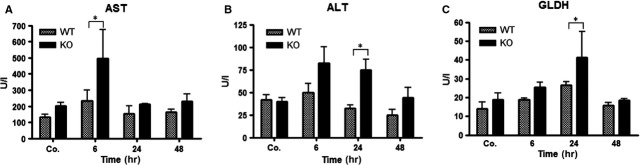
Measurement of aspartate transaminase (AST), alanine aminotransferase (ALT) and glutamate dehydrogenase (GLDH) concentrations after irradiation in mice serum of WT and PECAM-1KO mice. The data are compared between WT and KO mice and with sham irradiated controls. Results represent means ± SEM of five experiments; *P < 0.05, *n* = 5.

To examine liver damage, the serum levels of ALT, AST and GLDH were analysed after irradiation in WT and PECAM-1 KO mice. The serum activity of ALT, AST and GLDH was increased after 6 hrs till 24 hrs and decreased afterwards in WT and PECAM-1 KO mice when compared to sham-irradiated controls. Of note, there was a significant difference between the WT and PECAM-1 KO mice with respect to the level of these enzymes after irradiation. The activity of ALT, AST and GLDH in PECAM-1 KO mice was significantly higher than WT at 6 and 24 hrs after irradiation (Fig. 7A–C).

### Phosphorylation of STAT-3 in mice hepatic tissue after irradiation

**Figure 8:**
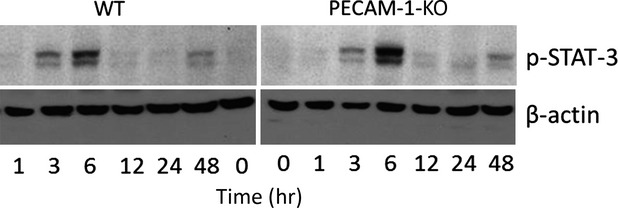
Western blot analysis of p-STAT3 in total protein from mice liver of wild-type (WT) and PECAM-1KO after liver irradiation at different time points. β-actin (∼42 kD) was used as a loading control. Results are representative of four to six experiments.

Signal transducer and activator of transcription-3 (STAT-3) is an important transcription factor in inflammatory signalling pathways. By means of Western blot using a specific antibody against p-STAT-3, phosphorylation of STAT-3 was detectable at 3 hrs with a further increase at 6 hrs in WT and PECAM-1 KO mice after irradiation. The maximum protein expression of p-STAT-3 was noticed at 6 hrs after irradiation in both animal groups; however, the band intensity was much higher in PECAM-1 KO mice than WT. Furthermore, a weak band was still obvious in KO-mice from 12 hrs until 48 hrs whereas such a change was only noticed at 48 hrs in WT-mice after irradiation (Fig. 8).

### Modulation of PECAM-1 and ICAM-1 protein expression, in cultured human monocytic cells U-937 after irradiation

**Figure 9:**
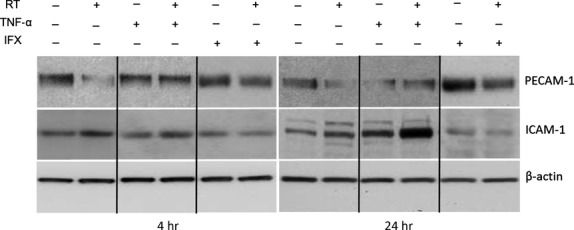
Detection of PECAM-1 and ICAM-1 by Western blot analysis of total protein from human U-937 cell line after treatment with tumour necrosis factor-alpha (TNF-α) or irradiation (RT) in the presence or absence of infliximab (IFX). β-actin (∼42 kD) was used as a loading control. Results are representative of four to six experiments.

To investigate the effect of irradiation in the presence of TNF-α/or anti-TNF-α on PECAM-1 and ICAM-1 gene regulation, human monocytic cells U-937 were irradiated in the presence/or absence of TNF-α and anti-TNF-α (IFX). A decrease in PECAM-1 with a parallel increase in ICAM-1 protein level was observed by Western blot analysis in human monocytic cells U-937 at 4 hrs after irradiation or TNF-α-administration as compared to the IFX- and non-treated control U-937 cells. Furthermore, reduction in PECAM-1 and rise in ICAM-1 protein concentration became more evident when irradiation and TNF-α were administered together at this time-point. Moreover, the most pronounced fall in PECAM-1 and peak in ICAM-1 protein expression was detected at 24 hrs after irradiation or/and TNF-α-administration. In contrast, the effect of irradiation was neutralized by addition of anti-TNF-α (IFX) to the culture medium together with irradiation (Fig. 9).

### Effect of RT on PECAM-1 and ICAM-1 by immunofluorescence double staining in the U937-cells

**Figure 10:**
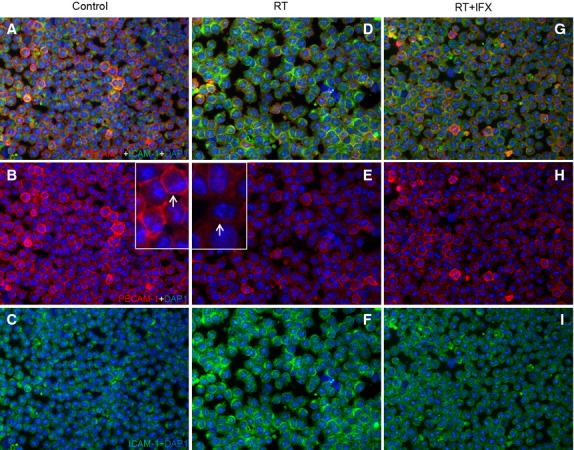
Double staining of U-937 cells sections with antibodies directed against PECAM-1 (green) and ICAM-1 (red), followed by fluorescence after 4 hrs of treatment. (A–C) control sections (D–F) RT (G–I) RT+IFX. Result show representative picture of four to six slides. Original magnification, ×100.

Using a monoclonal antibody directed against PECAM-1/CD-31, immunoreactivity of PECAM-1 was detectable in unstimulated and irradiated U-937 cells confirming the results obtained at protein level by Western blot with PECAM-1 and ICAM-1 antibody. A rapid and progressive reduction in PECAM-1 positivity in U-937 cells after irradiation was observed reaching a nadir of inhibition at 4 hrs compared to sham-irradiated control cells. An opposite trend was noticed in ICAM-1 expression. An increase in ICAM-1 was evident at 4 hrs after irradiation. Addition of anti-TNF-α into the medium inhibited the effect of irradiation on PECAM-1 and ICAM-1 (Fig. 10).

## Discussion

PECAM-1 belongs to the Ig superfamily of cell-adhesion molecules and is important for the modulation of inflammatory responses. Accordingly, a role of PECAM-1 as regulator of pro-inflammatory and anti-inflammatory functions both in leukocytes and endothelial cells has also been described. 34,38.

Previously, we could show that TNF-α reduced PECAM-1 (CD31) expression in parallel to ICAM-1 up-regulation in several acute inflammation models. This is important for the regulation of the recruitment and transmigration of inflammatory cells 26,28. The role of PECAM-1 in inflammation is controversially debated, and poorly understood in irradiation.

To investigate the role of PECAM-1 in radiation-induced liver damage, we analysed inflammatory mediators in WT and PECAM-1-KO mice. We found a reduction of PECAM-1 levels in parallel to a rise of ICAM-1 in WT mice after irradiation. ICAM-1 was further elevated in PECAM-1-KO mice after irradiation. Accordingly, irradiation showed a quick, early and transient recruitment of leucocytes (NG) in WT-mice. Interestingly, the number of recruited NG was much higher (around the portal area) in PECAM-1-KO-mice compared to WT, whilst no increase in mononuclear phagocytes (F4/80+ cells) was observed by immunofluorescence at any time-point after irradiation.

In accordance with the increased number of NG, a brisk elevation in gene expression of hepatic TNF-α, followed by CXC-chemokines (CXCL1, CXCL8) was measured in both animal groups, together with an increase in liver transaminases (ALT, AST, GLDH). Interestingly, the magnitude of increase in these cyto- and chemokines as well as of liver transaminases was significantly higher in KO-mice than in WT-mice after irradiation, indicating an important regulative function of PECAM-1 in radiation-induced hepatic damage.

Leucocyte migration from the blood to sites of injury is a central event in inflammation 9 and triggered by activation of cytokines and chemokines 4,7. Despite their role in tissue damage, both neutrophils and macrophages are also important in initiating tissue healing processes. This suggests a necessary balance between limiting inflammation and beginning of tissue repair.

In the liver, the process of leucocyte migration occurs through the portal vessel and/or through the sinusoidal wall. Our current study in mice showed recruitment of NGs around the portal area of the liver after irradiation. Our previous study in a comparable rat model of irradiation 32 using immunohistochemistry and laser capture microdissection allowed us to localize an increase in chemokines responsible for granulocyte recruitment in the portal area compared to the liver parenchyma. A similar behaviour can be supposed within the current study.

Another interesting aspect of the current study was the observation of a lack of increase in mononuclear phagocyte number after irradiation. Similarly, in a mouse model of irradiation, inhibition of monocyte entry into the optic nerve after irradiation has already been described 39. This study proposed a link between the inhibition of monocyte entry with increased gene expression of Glycosylation-dependent cell adhesion molecule-1 (GlyCAM-1). Although, the exact mechanism how irradiation inhibits the entry of monocyte into the liver, is not known yet, one can speculate that radiation acts on both sides, endothelial cells and leucocytes possibly including modification of CAM-expression and/or functional changes in mononuclear phagocytes. It means, regardless of their number, inactivation of mononuclear phagocyte function after irradiation could influence their normal defence activity and ultimately affects the normal tissue repair process.

Chemotactic cytokines, i.e. chemokines represent a family of chemotactic peptides which act on multicellular targets. In the liver, chemokines can be synthesized by hepatocytes, Kupffer cells, sinusoidal endothelium, cholangiocytes and stellate cells 40. Chemotactic signals include CXC chemokines such as CXCL1/KC and CXCL8/IL-8, which are potent chemoattractants for NG and their increased production causes NG infiltration and extravasation 3,7 which would be in accordance with our study.

In fact, production of inflammatory mediator(s) is essential for host defence, and neutrophils are the first line of immune defence 40,41. An unrestricted release of inflammatory mediators together with an increased inflammatory infiltrate could lead to tissue damage or severe inflammation.

To prevent an overshooting immune response, the balance of inflammatory signals needs to be restored. We propose PECAM-1 as a player for the maintenance of inflammatory homeostasis: PECAM-1 is involved in immune-cell transmigration. However, the lack of PECAM-1 exhibited prolonged and induced granulocyte infiltration together with an increased release of pro-inflammatory mediators in the liver in our irradiation model.

This implies a role of PECAM-1 not only in immune-cell infiltration but also in the termination of an overshooting immune response. Our results are in accordance with previous findings. As previously reported, PECAM-1 showed a protective role against endotoxin shock in mice 25. Similarly, lack of PECAM-1 resulted in amplified inflammation followed by liver injury in a nonalcoholic steatohepatitis mouse model 42.

Hepatocytes are the major liver cell type and main source of positive acute phase-proteins 43. The strong induction of these proteins in the current study can be caused by a direct effect of generated free radicals in the hepatocytes. Oxidative stress prompted signalling pathways could induce chemokine and cytokine synthesis as well as changes in regulation of adhesion molecules gene(s) 44. Mitochondrial reactive oxygen species (ROS) production, caused by radiation, can directly affect mitochondria 45, probably inducing hepatic injury 32. An increased ROS production in PECAM-1 deficient mice has already been reported 46. ROS can activate pro-inflammatory signalling pathways and promote migration 4.

Accordingly, we could show phosphorylation of the inflammation-associated transcription factor STAT-3 in both animal groups after irradiation, with an additional major increase in PECAM-1-KO mice compared to WT.

Ionizing radiation has further been shown to induce modifications of hepatic enzymatic activities implicated in bile acid biosynthesis 47. CK19 is expressed by cholangiocytes, and irradiation induced hepatic CK19 expression has already been reported 48. The external stress explains the increased number and size of bile ducts (CK 19 staining). This bile duct epithelia secretion could further increase the level of α-SMA in fibroblast or myofibroblast of the liver 49. Under stress circumstances, apart from Kupffer cells, a role of activated fibroblasts or myofibroblasts together with cholangiocytes in releasing the inflammatory mediators is well-established 32,49 which may elucidate the role of increased expression of CK-19 and α-SMA during radiation-induced acute stress conditions.

In our rat liver irradiation model, infiltration of NG and an increase in pro-inflammatory cytokines, mainly TNF-α was observed 32,37. Likewise, we observed an increase in TNF-α in the current study. Both, NG infiltration and TNF-α elevation were higher in PECAM-1 KO-mice. In fact, consideration of our data further implies that PECAM-1 could have a dual role: on one hand, PECAM-1 is the target of inflammatory cytokines, on the other hand it controls their production (*e.g*. TNF-α), with the aim to maintain inflammatory homeostasis.

To answer whether blocking radiation-induced TNF-α production by anti-TNF-α (IFX) could have an effect on PECAM-1 and ICAM-1 gene expression (reduction of pro-inflammatory conditions), a human monocytic cell line U-937 (characteristic of macrophages) was cultured and treated with TNF-α in the presence or absence of irradiation and IFX.

Similar to what we observed in the liver tissue after irradiation, a decline in protein expression of PECAM-1 was found in parallel to ICAM-1 induction after TNF-α or radiation treatment. This effect was suppressed by the addition of an anti-TNF-α antibody (IFX).

These findings were confirmed by immunofluorescence detection of PECAM-1-and ICAM-1-positive cells, which showed a complete recovery of PECAM-1 and a reduction of ICAM-1 after treatment with IFX and irradiation.

Several previous studies 5,6,29 showed that IFX exerts its anti-inflammatory effect by inhibiting TNF-α and thus minimizing inflammation and its mediators.

Based on these findings, our current study extends our previous knowledge and provides a clear insight that PECAM-1 KO mice are more sensitive to radiation-induced inflammation.

Compared to WT, lack of PECAM-1 induced a higher magnitude of NGs infiltration into the liver indicating a spontaneous migration of inflammatory cells. This migration was further enhanced after irradiation, accompanied by an increased expression of inflammatory cyto- (*e.g*. TNF-α), chemokines and probably through induction of other molecules such as CD99 or GlyCAM. The later could replace PECAM-1 in the current setting. This aspect is under further investigation currently.

Furthermore, hepatic damage was also increased in PECAM-1 KO mice when compared to WT. This confirms the importance of PECAM-1 down-regulation for radiation-induced inflammation.

Tumour necrosis factor-α is an important pro-inflammatory cytokine in radiation-mediated liver damage. Accordingly, anti-TNF-α therapy prevented the radiation-induced down-regulation of PECAM-1 expression *in vitro*. Therefore, neutralization of TNF-α using an anti-TNF-α antibody seems to be a strategy to prevent further radiation damage.

Influx of inflammatory cells from the circulation into the area of inflammation is regulated by cell-adhesion molecules and chemokines. These molecules serve as stimulating targets for both acute and chronic inflammation.

## Supporting Information

**Table S1** CT-values of GAPDH, PECAM-1 and ICAM-1in WT and PECAM-1 KO mice liver after irradiation.

**Table S2** CT-values of cytokine and chemokines in WT and PECAM-1 KO mice liver after irradiation.
