# Supplementary material for: Role of PECAM-1 in radiation-induced liver inflammation
Source: J Cell Mol Med. 2015 Jul 14;19(10):2441–52. doi: 10.1111/jcmm.12630 (PMC4594685; doi:10.1111/jcmm.12630)
Supplement: Supplementary file 1 [file jcmm0019-2441-sd1.docx]

**Supplementary Tables:**

**Supplementary Table 1:** CT-values of GAPDH, PECAM-1 and ICAM-1 in WT and PECAM-1 KO mice liver after irradiation

| Time (h) | GAPDH (WT) | GAPDH (KO) | PECAM-1 (WT) | PECAM-1 (KO) | ICAM-1 (WT) | ICAM-1 (KO) |
| --- | --- | --- | --- | --- | --- | --- |
|  |  |  |  |  |  |  |
| Control | 19.1±0.4 | 19.96±0.15 | 27.81±0.06 | No detection | 29.11±0.16 | 29.16±0.4 |
| 1 | 19.43±0.04 | 19.53±0.2 | 28.97±0.18 |  | 27.86±0.43 | 26.93±0.5 |
| 3 | 19.39±0.12 | 19.49±0.22 | 28.80±0.26 |  | 27.89±0.55 | 26.88±0.5 |
| 6 | 19.65±0.11 | 19.22±0.3 | 29.90±0.14 |  | 27.60±0.3 | 25.92±0.6 |
| 12 | 19.43±0.27 | 19.1±0.13 | 29.02±0.22 |  | 28.46±0.27 | 28.64±0.2 |
| 24 | 19.36±0.14 | 19.52±0.11 | 28.30±0.23 |  | 28.15±0.08 | 27.89±0.3 |
| 48 | 19.49±0.25 | 19.82±0.18 | 28.45±0.24 |  | 28.39±0.3 | 27.75±0.3 |

**Supplementary Table 2:** CT-values of cytokine and chemokines in WT and PECAM-1 KO mice liver after irradiation

| Time (h) | TNF-α (WT) | TNF-α (KO) | CXCL1 (WT) | CXCL1 (KO) | CXCL8 (WT) | CXCL8 (KO) |
| --- | --- | --- | --- | --- | --- | --- |
|  |  |  |  |  |  |  |
| Control | 35.04±0.55 | 35.57±0.7 | 29.38±0.31 | 28.12±0.1 | 29.88±0.12 | 28.87±0.35 |
| 1 | 33.44±0.05 | 33.54±0.07 | 29.54±0.19 | 28.6±.03 | 29.91±0.01 | 28.47±0.16 |
| 3 | 33.32±0.55 | 31.37±0.85 | 24.37±1.28 | 24.23±0.37 | 25.01±0.16 | 25.41±0.16 |
| 6 | 33.96±0.18 | 34.01±0.15 | 24.41±0.74 | 21.91±0.48 | 23.3±0.23 | 21.92±0.31 |
| 12 | 34.04±0.36 | 34.71±0.12 | 26.71±0.63 | 27.8±0.17 | 28.1±0.2 | 28.33±0.08 |
| 24 | 33,54±0.22 | 34.3±0.66 | 26.95±0.97 | 25.4±0.6 | 25.78±0.08 | 25.21±0.4 |
| 48 | 34.11±0.84 | 33.22±0.4 | 24.27±0.45 | 24.17±0.16 | 25.28±0.15 | 24.65±0.3 |
